# Social media use and child cigarette smoking and e-cigarette use: A cohort study 2015–2023

**DOI:** 10.18332/tid/211432

**Published:** 2025-11-20

**Authors:** Anthony A. Laverty, Jennie C. Parnham, Martin McKee, Filippos T. Filippidis, Nicholas S. Hopkinson

**Affiliations:** 1 Public Health Policy Evaluation Unit, School of Public Health, Imperial College London, London, United Kingdom; 2 Department of Health Services Research and Policy, London School of Hygiene & Tropical Medicine, London, United Kingdom; 3 National Heart and Lung Institute, Imperial College London, London, United Kingdom

**Keywords:** children, smoking, tobacco use, electronic nicotine delivery systems, vaping

## Abstract

**INTRODUCTION:**

There are growing concerns that advertising and promotion on social media are driving youth use of tobacco and e-cigarettes. The UK provides an instructive example as it has high levels of e-cigarette use, high levels of social media use and a restrictive tobacco control environment. Existing evidence in the UK, however, has not focused on children, and has not been updated to reflect changes in patterns of social media use and in the use of these products. The aim of this study is to assess the associations of social media use with smoking and vaping.

**METHODS:**

Using data from the United Kingdom Household Longitudinal Study on adolescents aged 10–17 years between 2015–2023, we employed generalized estimating equation (GEE) models to estimate the relationships between time spent on social media and likelihood of smoking tobacco and using e-cigarettes. Models were controlled for possible confounders including sociodemographics and whether children lived in a home with e-cigarette use or tobacco smoking. We included data from 9359 participants with 25704 observations.

**RESULTS:**

Current cigarette smoking was reported by 4.9% of the sample and current e-cigarette use by 3.1%. Our adjusted models found strong relationships between time spent on social media and both smoking and vaping (p for trend <0.001). For example, use of social media for ≥7 hours/day was linked to greater odds of tobacco (adjusted odds ratio, AOR=5.13; 95% CI: 3.32–7.95) and e-cigarette use (AOR=4.26; CI: 2.25–8.08).

**CONCLUSIONS:**

This study finds associations between time spent on social media and both smoking and vaping among children. Enforcing regulations on content and restricting the duration of social media use may be warranted to protect children’s health.

## INTRODUCTION

Social media use is now near ubiquitous among young people, and represents a potential novel vector for negative health behaviours^1^. As tobacco dependence mostly begins in childhood^2^, this is a potential concern, given that a systematic review identified links between exposure to advertising on social media and tobacco smoking and e-cigarette use^3^. Most studies were in the US, with one from each of India, Australia and Indonesia. Three existing studies have studied the link between social media and smoking/e-cigarette use in the UK. One, using data until 2017, found that social media use at the age of 14 years was linked to later e-cigarette use^4^. One cross national analysis included the UK and identified links between social media and substance use, but did not disaggregate different products^5^. A third study^6^ used a national cohort to examine this issue until 2021 among those aged 10–25 years, i.e. prior to the sharp increase in youth vaping in 2022.

Approximately 10% of UK adults vape, while 7% of children were regular vapers in 2025 and 20% report having ever tried vaping^7,8^. Tobacco cannot legally be sold to those aged under 18 years and neither can e-cigarettes which contain nicotine. As social media platforms continue to evolve, as does tobacco and e-cigarette use, in particular disposable devices, we aim to investigate the association of social media use with tobacco and e-cigarette use among UK children up until 2023.

## METHODS

We used data for adolescents aged 10–17 years from Understanding Society [the UK Household Longitudinal Study (UKHLS)]. Sampling is designed to be nationally representative and the original sample at baseline was based on a stratified probability sample of 28000 households^9^. Participants are followed up annually. Data are collected by face-to-face interviews and online with self-completed questions. Standardized questionnaires are administered with data collected electronically (i.e. computer-assisted personal interviewing). No specific tools were used to collect social media use data which were based on self-report; those aged 10–15 years complete a shorter questionnaire with permission from caregivers and those aged 16–17 years are interviewed.

We analyzed data from 2015–2016 to 2022–2023. Outcomes were current cigarette smoking and current e-cigarette use (both defined as use at least once per week). Exposure was assessed with the question: ‘On a normal week day, that is Monday to Friday, how many hours do you spend chatting or interacting with friends through social media, gaming websites or apps?’ with responses ‘none or not a member’, ‘less than an hour’, ‘1–3 hours’, ‘4–6 hours’ and ‘≥7 hours’. Possible confounders were age (continuous), sex (binary), UK nation (England, Scotland, Wales, Northern Ireland), ethnicity (White, non-White), household income in three groups, and parental tobacco or e-cigarette use. Model building was based on prior research and data availability. All tests were two tailed with a statistical significance threshold of p<0.05 and analyses were conducted in Stata.

### Statistical analysis

We used generalized estimating equation (GEE) models (family: binomial; link: logit; correlation matrix: exchangeable) which take into account repeated observations from the same people and changes over time^10^. We used GEE models rather than mixed models to focus on population level impacts and an exchangeable correlation structure to deal with within-subject correlation. Analyses used UKHLS survey weights, which account for sampling and non-response biases. We present tests for trend based on social media use frequency. We also present sex-stratified models as well as interactions of parental smoking/vaping and social media use.

## RESULTS

The sample contained 25704 observations from 9358 individual participants. In the whole sample, 4.9% reported current cigarette smoking for at least one data point, and 3.1% reported current e-cigarette use.

Data broken down by categories of social media use are given in Supplementary file Table 1. These show, for example that there were lower proportions of males using social media ≥7 hours/day than none or not at all (39.0% vs 56.6%). Mean ages were also higher among those using social media ≥7 hours/day (mean=15.47 years, SD=1.71) than those using social media none or not at all (mean=11.6 years, SD=1.7). Outcomes by year are shown in Supplementary file Figure 1, which shows an increase in e-cigarette use in 2023. Outcomes by age are shown in Supplementary file Figure 2.

Our adjusted GEE analyses showed current tobacco smoking to be more common among those using social media more frequently (p for trend <0.001) (Table 1). Use of social media for <1 hour a day was associated with over twice the odds of current smoking (AOR=1.92; 95% CI: 1.29–2.86), while use for ≥7 hours/day was linked to an even greater increase (AOR=5.13; 95% CI: 3.32–7.95) compared to non-use.

**Table 1:** Associations, from a generalized estimating equation (GEE) model, of social media use with current cigarette smoking in the UK, 2015–2023 (N=6624)

*Variables*	*AOR (95% CI)*	*p*
**Weekday social media use** (hours/day)		
None or not member ®	1	
<1	1.92 (1.29–2.86)	0.001
1–3	3.13 (2.16–4.56)	<0.001
4–6	3.91 2.61–5.87)	<0.001
≥7	5.13 (3.32–7.95	<0.001
**Year**		
2015–2016 ®	1	
2016–2017	1.41 (1.12–1.77)	0.003
2017–2018	1.26 (0.98–1.62)	0.069
2018–2019	1.73 (1.34–2.24)	<0.001
2019–2020	1.17 (0.87–1.59)	0.300
2020–2021	1.12 (0.80–1.57)	0.513
2021–2022	0.86 (0.59–1.25)	0.436
2022–2023	1.31 (0.90–1.91)	0.165
**Sex** (Female vs Male)	1.03 (0.84–1.27)	0.744
**Age** (years)	1.26 (1.21–1.32)	<0.001
**UK nation**		
England ®	1	
Wales	0.53 (0.33–0.86)	0.011
Scotland	0.73 (0.49–1.10)	0.132
Northern Ireland	0.93 (0.62–1.41)	0.745
**Rural area vs Urban**	1.01 (0.80–1.29)	0.922
**Non-White ethnicity** (vs White)	0.72 (0.52–1.01)	0.059
**Household income group**		
Lowest ®	1	
Middle	0.79 (0.66–0.95)	0.014
Highest	0.65 (0.52–0.82)	<0.001
**Parents smoking/vaping status**		
Not smoking nor vaping ®	1	
Smoking only	2.81 (2.22–3.56)	<0.001
Vaping only	2.22 (1.57–3.15)	<0.001
Both smoking and vaping	2.07 (1.44–2.95)	<0.001

AOR: adjusted odds ratio. All covariates shown here included in models. ® Reference categories.

In GEE analyses, current vaping was more likely among more frequent social media users (p for trend <0.001) (Table 2). Use of social media for <1 hour/ day was associated with greater odds of current e-cigarette use (AOR=1.89; 95% CI: 1.08–3.32) and use for ≥7 hours/day was linked to a 4-fold increase (AOR=4.26; 95% CI: 2.25–8.08) compared to no social media use.

**Table 2:** Associations, from a generalized estimating equation (GEE) model, of social media use with current e-cigarette use in the UK, 2015–2023 (N=6549)

*Variables*	*AOR (95% CI)*	*p*
**Weekday social media use** (hours/day)		
None or not member ®	1	
<1	1.89 (1.08–3.32)	0.027
1–3	2.41 (1.40–4.16)	0.002
4–6	3.94 (2.23–6.97)	<0.001
≥7	4.26 (2.25–8.08)	<0.001
**Year**		
2015–2016 ®	1	
2016–2017	0.42 (0.30–0.60)	<0.001
2017–2018	0.47 (0.32–0.68)	<0.001
2018–2019	0.70 (0.51–0.98)	0.037
2019–2020	0.66 (0.44–0.99)	0.047
2020–2021	0.80 (0.53–1.23)	0.310
2021–2022	1.37 (0.95–1.96)	0.091
2022–2023	2.58 (1.86–3.58)	<0.001
**Sex** (Female vs Male)	1.49 (1.17–1.89)	0.001
**Age** (years)	1.47 (1.39–1.56)	<0.001
**UK nation**		
England ®	1	
Wales	0.68 (0.39–1.18)	0.170
Scotland	0.77 (0.50–1.19)	0.234
Northern Ireland	0.82 (0.50–1.33)	0.415
**Rural area vs Urban**	1.06 (0.81–1.39)	0.682
**Non-White ethnicity** (vs White)	0.62 (0.39–0.96)	0.033
**Household income group**		
Lowest ®	1	
Middle	0.81 (0.63–1.06)	0.121
Highest	0.86 (0.65–1.13)	0.286
**Parents smoking/vaping status**		
Not smoking nor vaping ®	1	
Smoking only	2.21 (1.61–3.04)	<0.001
Vaping only	3.04 (2.10–4.40)	<0.001
Both smoking and vaping	1.78 (1.14–2.77)	0.010

AOR: adjusted odds ratio. All covariates shown here included in models. ® Reference categories.

**Figure 1:**
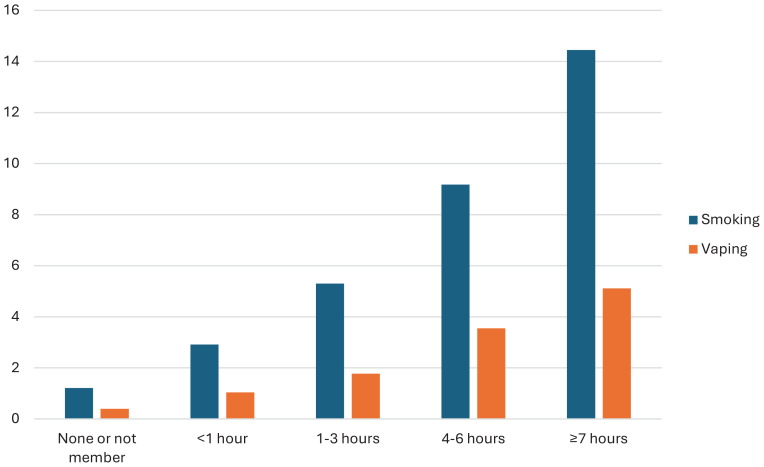
Tobacco smoking and e-cigarette use (%) by daily use of social media 2015–2023

Interaction by sex was statistically significant (p<0.001), and stratified models found larger AOR point estimates for girls than boys for all categories of social media use and both outcomes (Supplementary file Table 2). For example, the AOR for tobacco smoking for ≥7 hours/day versus none was 5.83 (95% CI: 3.19–10.67) for girls and 4.80 (95% CI: 2.59–8.92) for boys. Differences were larger for e-cigarettes: the AOR for e-cigarette use ≥7 hours/ day versus none was 7.45 (95% CI: 2.27–24.40) for girls and 4.04 (95% CI: 1.84–8.90) for boys.

## DISCUSSION

The main finding of this study is a strong association between social media use and the likelihood of both smoking and vaping among children. This was independent of a range of sociodemographic factors including household income and product use by parents. The period of the study coincides with marked increases both in youth vaping and social media use^11^ and the association appeared to be more pronounced in girls than boys.

Increased exposure to advertising and promotion very likely explains part of this association. Vaping and tobacco content are widespread on social media and they are more likely to be portrayed positively than not^12-14^. Evidence on social media impacts on smoking and vaping is growing, with experimental evidence finding short-term exposure to social media materials increases intention to vape, while longitudinal evidence links social media to starting smoking^15,16^. Our analysis among children finds larger effect sizes than previously observed in adults^6^, but we cannot say if this is due to targeted marketing or greater susceptibility as we do not have information on the social media platforms children were using or what they viewed. We cannot, therefore, provide the more granular assessment needed to assess the effects of different platforms. Social media use itself has addictive elements^17^, and elucidation of the precise pathways between social media and smoking/vaping should be a priority for future research.

### Limitations

In addition to being unable to assess more granularly which social media platforms were used there are other limitations to this study. All data are based on self-reports, and while we have controlled for a range of potentially relevant factors, other aspects were unable to be studied here. These include the roles of peers both online and face to face. Other factors include the role of mental health or smoking and vaping by peers and siblings. While we have used data from a nationally representative cohort with survey weights for non-response, the generalizability to outside the UK is difficult to assess. Finally, the impacts of the changing landscape of tobacco and vapes in the UK mean that similar research should be conducted in the future to assess if relationships change.

### Implications

This study adds to evidence that social media may be an important determinant of health. Regulatory action and other steps to implement the WHO Framework Convention on Tobacco Control measures that prevent tobacco industry advertising, promotion and sponsorship (TAPS) via social media, are crucial. The strong association between social media use and smoking and vaping among children underlines the need to make social media a safer environment for young users. Importantly, as this study focuses on children, who should not be able to purchase tobacco or e-cigarettes legally, the high levels of use of tobacco and e-cigarettes here, point to issues in enforcing laws on age of sale.

## CONCLUSIONS

This study reinforces concerns about links between social media use and smoking and vaping among children. In April 2025 the UK’s media regulator, Ofcom, published guidance on protecting children from online harm, including harmful substances^18^. It will be essential to continue to monitor the impacts of social media on a range of health outcomes, including tobacco and e-cigarette use. Although research evidence is still emerging, steps to reduce the duration of social media use may also be prudent on health grounds.

## Supplementary Material



## Data Availability

The data supporting this research are available from the following sources: https://ukdataservice.ac.uk
