# Supplementary material for: Social media use and child cigarette smoking and e-cigarette use: A cohort study 2015–2023
Source: Tob Induc Dis. 2025 Nov 20;23:10.18332/tid/211432. doi: 10.18332/tid/211432 (PMC12641251; doi:10.18332/tid/211432)
Supplement: Supplementary file 1 [file TID-23-178-s1.pdf]

Supplementary file

Supplementary Figure 1

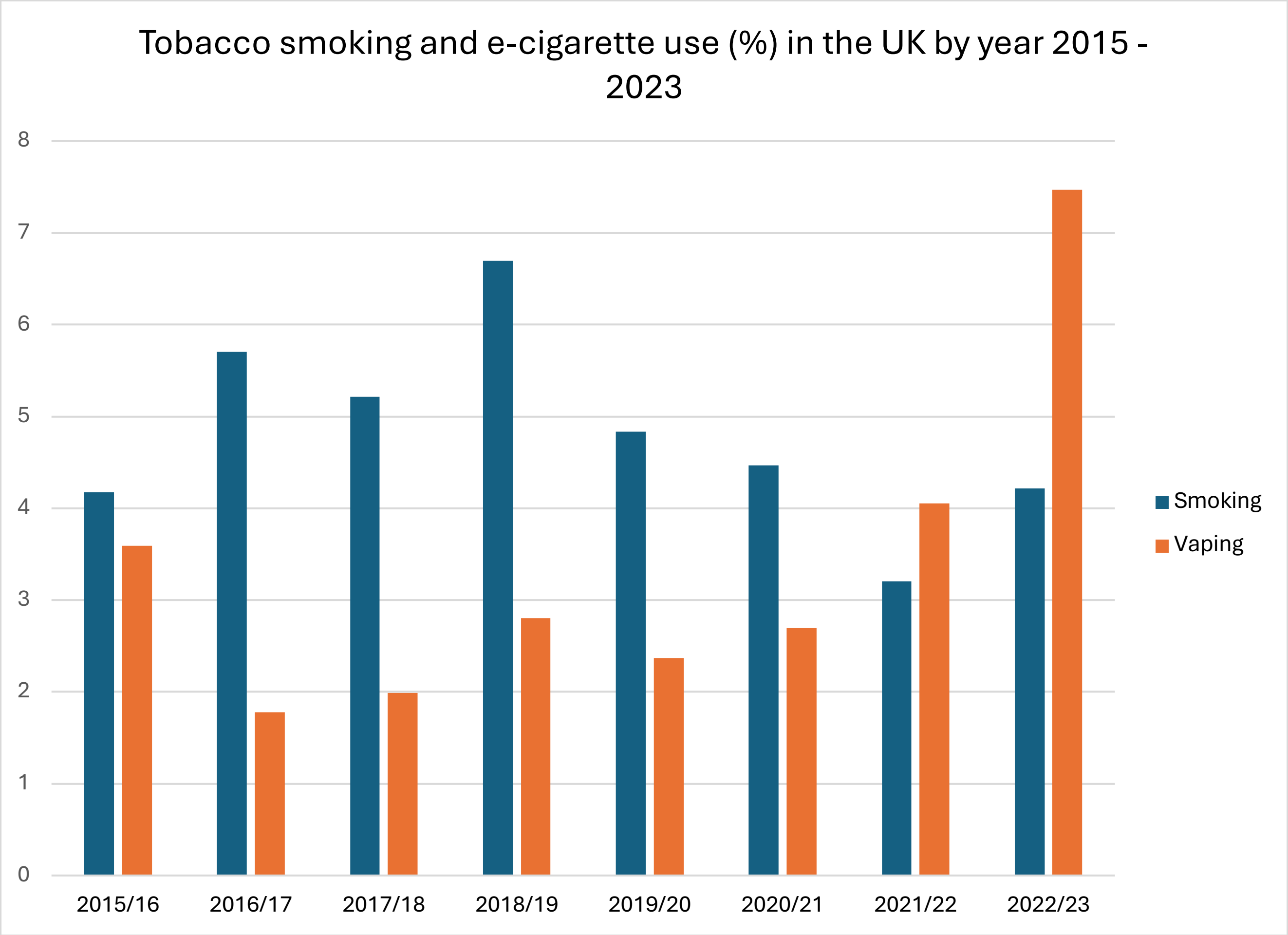

Supplementary Figure 2

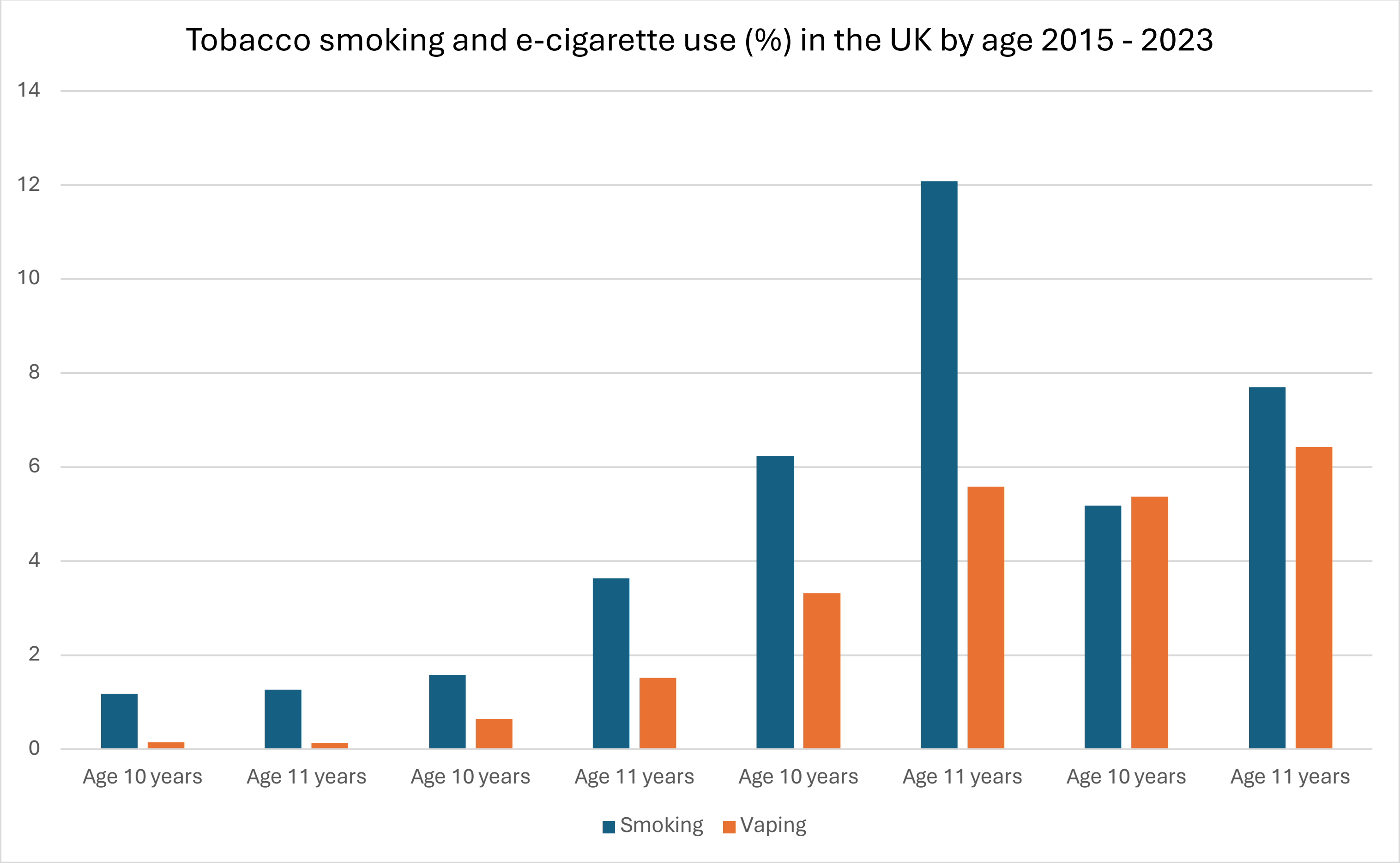

Supplementary Table 1: Description of sample observations by social media use in the UK 2015-2023

Social media use (hours/daily)	None or not member	<1 hour	1-3 hours	4-6 hours	≥7 hours	P value for difference between groups
Current cigarette smoking (%)	1.2	2.9	5.3	9.2	14.4	<0.001
Current e-cigarette use (%)	0.4	1.0	1.8	3.5	5.1	<0.001
Male (%)	56.6	51.8	46.1	39.2	39.0	<0.001
Mean age (SD)	11.6 (1.7)	13.1 (2.05)	14.00 (2.08)	14.75 (1.93)	15.5 (1.71)	<0.001
Non-White ethnicity (%)	34.3	31.1	25.2	22.3	23.8	<0.001
Non-Urban areas (%)	20.7	22.0	23.6	22.6	21.9	0.004
Mean Household income (monthly £) (SD)	1692 (3334)	1719 (1344)	1668 (982)	1612 (1071)	1504 (870)	<0.001
Parental cigarette smoking (%)	16.0	15.5	17.8	21.6	25.8	<0.001
Parental e-cigarette use (%)	7.4	7.4	8.6	11.5	11.2	<0.001

SD: Standard Deviation

Supplementary Table 2: Associations of social media use with current cigarette smoking and current e-cigarette use from Generalised Estimating Equation models and UKHLS in the UK 2015-2023

Weekday social media use (hours/day)	AOR	p value	Lower CI	Upper CI
None or not member	ref	ref	ref	ref
<1 hour	1.92	0.001	1.29	2.86
1-3 hours	3.13	<0.001	2.16	4.56
4-6 hours	3.91	<0.001	2.61	5.87
≥7 hours	5.13	<0.001	3.32	7.95
Year 2015/2016	ref	ref	ref	ref
2016/17	1.41	0.003	1.12	1.77
2017/18	1.26	0.069	0.98	1.62
2018/19	1.73	<0.001	1.34	2.24
2019/20	1.17	0.300	0.87	1.59
2020/21	1.12	0.513	0.80	1.57
2021/22	0.86	0.436	0.59	1.25
2022/23	1.31	0.165	0.90	1.91
Sex (referencemale)	1.03	0.744	0.84	1.27
Age (in years)	1.26	<0.001	1.21	1.32
England	ref	Ref	ref	ref
Wales	0.53	0.011	0.33	0.86
Scotland	0.73	0.132	0.49	1.10
Northern Ireland	0.93	0.745	0.62	1.41
Rural area vs. urban	1.01	0.922	0.80	1.29
Non-white ethnicity (vs. white)	0.72	0.059	0.52	1.01
Lowest household income group	ref	Ref	ref	ref
Middle household income group	0.79	0.014	0.66	0.95
Highest household income group	0.65	<0.001	0.52	0.82
Parents not smoking nor vaping	ref	Ref	ref	ref
Parents smoking only	2.81	<0.001	2.22	3.56
Parents vaping only	2.22	<0.001	1.57	3.15
Parents both smoking and vaping	2.07	<0.001	1.44	2.95

AOR = adjusted odds ratio, CI = confidence interval, ref = reference

Supplementary Table 3: Associations of social media use with current e-cigarette use from Generalised Estimating Equation model and UKHLS in the UK 2015-2023

Weekday social media use (hours/day)	AOR	p value	Lower CI	Upper CI
None or not member	ref	ref	ref	ref
<1 hour	1.89	0.027	1.08	3.32
1-3 hours	2.41	0.002	1.40	4.16
4-6 hours	3.94	<0.001	2.23	6.97
≥7 hours	4.26	<0.001	2.25	8.08
year 2015/2016	ref	ref	ref	ref
2016/17	0.42	<0.001	0.30	0.60
2017/18	0.47	<0.001	0.32	0.68
2018/19	0.70	0.037	0.51	0.98
2019/20	0.66	0.047	0.44	0.99
2020/21	0.80	0.310	0.53	1.23
2021/22	1.37	0.091	0.95	1.96
2022/23	2.58	<0.001	1.86	3.58
Sex (referencemale)	1.49	0.001	1.17	1.89
Age (in years)	1.47	<0.001	1.39	1.56
England	ref	ref	ref	ref
Wales	0.68	0.170	0.39	1.18
Scotland	0.77	0.234	0.50	1.19
Northern Ireland	0.82	0.415	0.50	1.33
Rural area vs. urban	1.06	0.682	0.81	1.39
Non-white ethnicity (vs. white)	0.62	0.033	0.39	0.96
Lowest household income group	ref	ref	ref	ref
Middle household income group	0.81	0.121	0.63	1.06
Highest household income group	0.86	0.286	0.65	1.13
Parents not smoking nor vaping	ref	ref	ref	ref
Parents smoking only	2.21	<0.001	1.61	3.04
Parents vaping only	3.04	<0.001	2.10	4.40
Parents both smoking and vaping	1.78	0.010	1.14	2.77

Supplementary Table 4: Associations of social media use with current e-cigarette and cigarette use from gender stratified Generalised Estimating Equation models. Data from UK Household Longitudinal Study 2015-2023.

Current cigarette use*								
	Males				Females			
**Daily social media use (hours/day)**	AOR	P value	Lower CI	Upper CI	AOR	P value	Lower CI	Upper CI
**None or not a member**	ref	p value	ref	ref	ref	ref	ref	ref
**Less than an hour**	1.84	0.031	1.06	3.19	2.03	0.013	1.16	3.55
**1-3 hrs**	3.02	<0.001	1.79	5.12	3.32	<0.001	1.98	5.56
**4-6 hrs**	3.76	<0.001	2.14	6.60	4.25	<0.001	2.40	7.51
**7 or more**	4.80	<0.001	2.59	8.92	5.83	<0.001	3.19	10.67
**Current e-cigarette use****								
**None or not a member**	ref		ref	ref	ref	ref	ref	ref
**Less than an hour**	1.62	0.166	0.82	3.22	2.95	0.053	0.99	8.80
**1-3 hrs**	2.05	0.033	1.06	3.94	4.08	0.011	1.38	12.06
**4-6 hrs**	3.72	<0.001	1.86	7.44	6.4	0.001	2.10	19.47
**7 or more**	4.04	0.001	1.84	8.90	7.45	0.001	2.27	24.40

Results from models controlled for year, age, sex, country in UK, self-defined ethnic group (reference category: White), an indicator of living in an urban or rural area, equivalised household net income and parental smoking and e-cigarette use. AOR = adjusted odds ratio, CI = confidence interval
* p value for interaction p<0.001 ** p value for interaction p<0.001

© 2025 Laverty A.A. et al.
